# Supplementary figures and images for: The differentiation state of the Schwann cell progenitor drives phenotypic variation between two contagious cancers
Source: PLoS Pathog. 2021 Nov 15;17(11):e1010033. doi: 10.1371/journal.ppat.1010033 (PMC8629380; doi:10.1371/journal.ppat.1010033)

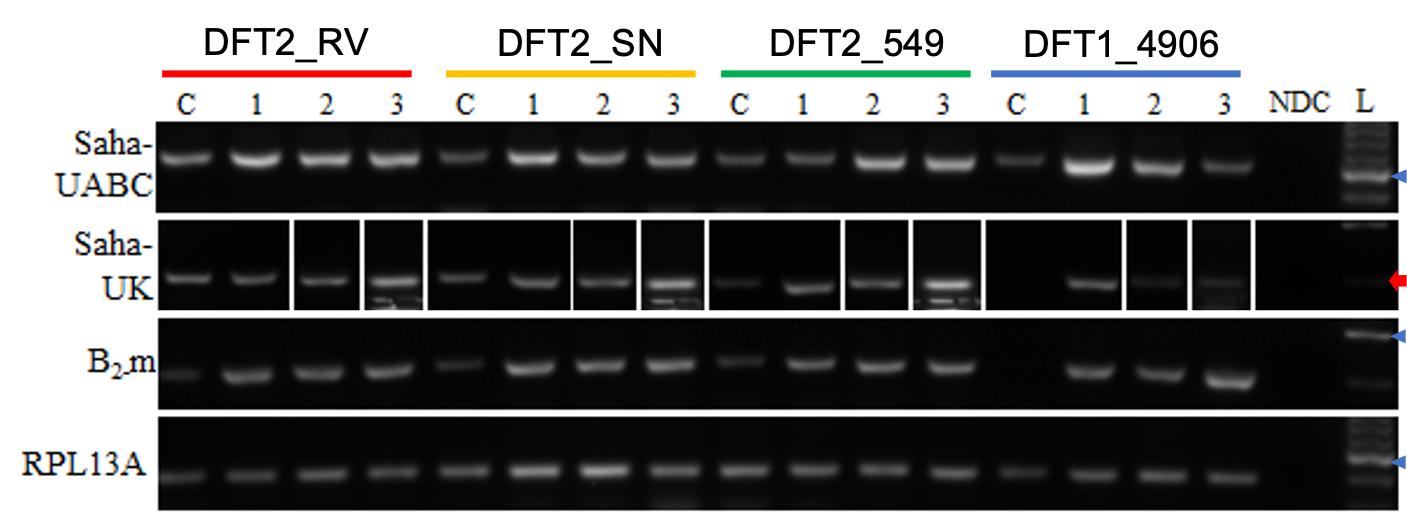

Supplement: S1 Fig — Agarose gels showing RT-PCR results for MHC class I associated genes on three independent biological replicates of DFT1_4906 and DFT2_RV, DFT2_SN and DFT2_549 treated with recombinant devil IFNγ in vitro. C indicates control cells grown in normal cell culture media, γ indicates cells treated for 16 hours with recombinant devil IFNγ. ND indicates a no cDNA negative control. L indicates a DNA ladder containing fragments of known size. Key molecular weights are indicated by blue arrowheads (300 bp) and red arrows (200 bp). RPL13A is a housekeeping gene used to control for the amount of cDNA in each PCR reaction. Related to Fig 3C. (TIF) [file ppat.1010033.s001.tif]

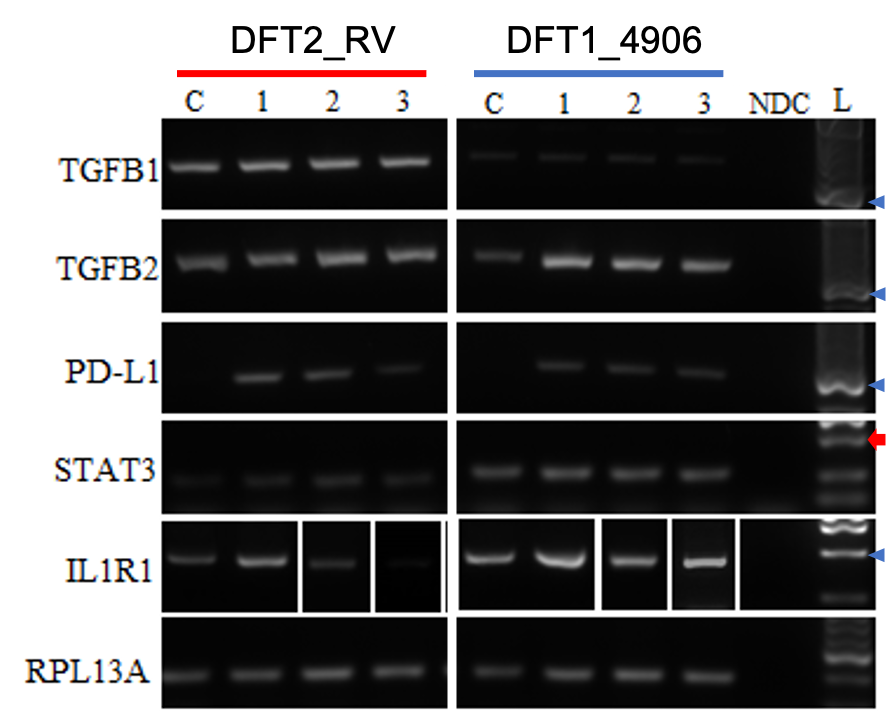

Supplement: S2 Fig — Agarose gels showing RT-PCR results for a panel of genes associated with immune function on three independent biological replicates of DFT1_4906 and DFT2_RV cell lines treated with recombinant devil IFNγ in vitro. C indicates control cells grown in normal cell culture media, γ indicates cells treated for 16 hours with recombinant devil IFNγ. ND indicates a no cDNA negative control. L indicates a DNA ladder containing fragments of known size. Key molecular weights are indicated by blue arrowheads (300 bp) and red arrows (200 bp). RPL13A is a housekeeping gene used to control for the amount of cDNA in each PCR reaction. Related to Fig 3E. (TIF) [file ppat.1010033.s002.tif]

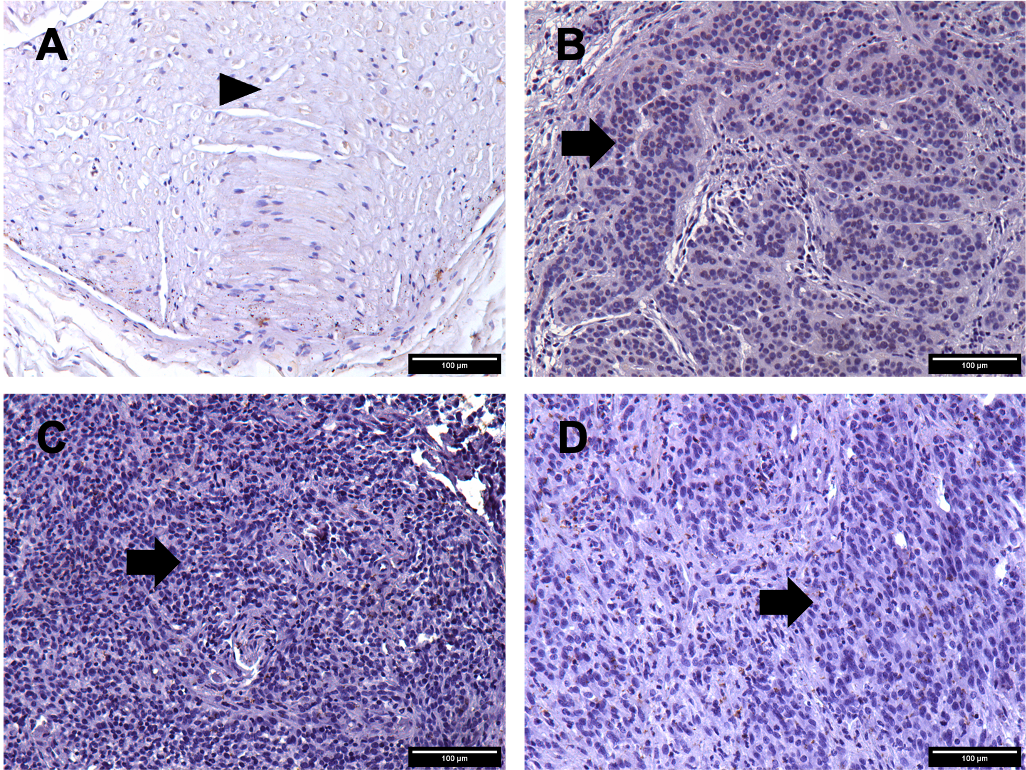

Supplement: S3 Fig — All images taken at 200X magnification. Scale bars indicate 100 μM. Black arrows indicate tumour cells, and black arrowheads indicate Schwann cells which are shown at a higher magnification in Fig 4. Blue staining indicates nuclei counterstained with haematoxylin. A) Tasmanian devil sciatic nerve, B) DFT1 tumour (Falestinya T1), C) DFT2 tumour (812 T1), D) DFT2 tumour (547 T1). Related to Fig 4. (TIF) [file ppat.1010033.s003.tif]
