# Supplementary material for: The differentiation state of the Schwann cell progenitor drives phenotypic variation between two contagious cancers
Source: PLoS Pathog. 2021 Nov 15;17(11):e1010033. doi: 10.1371/journal.ppat.1010033 (PMC8629380; doi:10.1371/journal.ppat.1010033)
Supplement: S8 Table — (DOCX) [file ppat.1010033.s011.docx]

| Gene | Sequences 5’-3’ | Annealing temperature (^o^C) | Amplicon length (bp) | Source |
| --- | --- | --- | --- | --- |
| RPL13A | F - ACAAGACCAAGCGAGGCCAGG  R - GCCTGGTATTTCCAGCCAACCTCA | 60 | 189 | Siddle et al. PNAS (2013) [1] |
| MAG | F - CGATTCGATTTTCCCGATGAGC  R - GTATTTGATGTCCAAGCTGGCG | 59 | 587 | This paper |
| MBP | F - GGATCAAAGTACCTGGCAACTGCA  R - CCTTGCCTGAGGCCCTCCTGGGCA | 60 | 144 | Murchison et al. Science (2010) [2] |
| MPZ | F - GTGGTTTACACGGACAGGGAG  R - CTTTCCACCGAGGATCCCCAA | 57 | 234 | This paper |
| PLP1 | F - TTCCCCAGCAAGACCTCAGC  R - GCCCATGAGTTTCAGGACAGC | 59 | 260 | This paper |
| PMP22 | F - CAATGTCCAGCACTGTTTCTCATCCTCTA  R - GAACAGGAACAAGGAAAGGACGCTGAA | 65 | 105 | Murchison et al. Science (2010) [2] |
| PRX | F - AACTGCCCACCATCAAGATGCC  R - TTCTGGGATGGACACTTCAGGC | 57 | 300 | This paper |
| FABP7 | F - ATTCTAAACCTCAGGGCCAG  R - TCCTGATCACCACTTTGTCC | 57 | 243 | This paper |
| L1CAM | F - TTGTGCAACCCCGAATCTACTG  R - AAACCTTCAGGCAGGAGCAAGC | 62 | 252 | This paper |
| NGFR | F - CCTACGGATACTACCAAGACG  R - GCCCAATAAAGGTGTGGTCCG | 58 | 286 | This paper |
| PDGFRA | F - CTCCAGAGCTTGAAGTTGAG  R - GTTTCCTTGGTAGCATGACG | 58 | 264 | This paper |
| TGFB1 | F - AAATTTGGCAACGGCTCCTGGCG  R - CGAAAATCGATGTAGAGCTGCCG | 63 | 388 | This paper |
| TGFB2 | F - CGACATGGATCAGTTCATGC  R - CCTTCACCAAGTTGGAAGC | 57 | 352 | This paper |
| PD-L1 | F - GTATTATTGGTTACGGGGGAGC  R - TGTGGCATTGACCCTGAGAGTGC | 62 | 259 | This paper |
| STAT3 | F - CAACTACAAGACCCTGAAAAGTCAAGGAGA  R - AGCATCTGTTCCTAGCTGCTGCATCTT | 65 | 100 | Murchison et al. Science (2010) [2] |
| IL1R1 | F - TTGCTGTTCGTCACAGTAGC  R - ATTGCCAAGGAGAGGTTTGC | 57 | 391 | This paper |
| Classical MHC class I heavy chains (Saha-UA/UB/UC, UABC) | F - CCGTGGGCTACGTGGACGA  GTCGTAGGCGAACTGAAG | 60 | 296 | Caldwell et al. (2018) [3] |
| Non-Classical MHC class I heavy chain Saha-UK (UK) | F - TGGTTGGACAAGAGTAA  R - CCTCAGGAAGATCCAGTCGTAAGTC | 60 | 190 | Caldwell et al. (2018) [3] |
| Non-Classical MHC class I heavy chain Saha-UD (UD) | F - ATGGAGAATGTGGACCGGGAC  R - TGAGTTCACTGCCTCATTCACT | 59 | 275 | Caldwell et al. (2018) [3] |
| B_2-_m | F - TGTGCATCCTTCCCTACCTGGAGG  R - CATTGTTGAAAGACAGATCGGACCGC | 60 | 300 | Siddle et al. (2013) [1] |

References

1. Siddle H V, Kreiss A, Tovar C, Yuen CK, Cheng Y, Belov K, et al. Reversible epigenetic down-regulation of MHC molecules by devil facial tumour disease illustrates immune escape by a contagious cancer. Proc Natl Acad Sci. 2013;110: 5103–5108. doi:10.1073/pnas.1219920110
2. Murchison EP, Tovar C, Hsu A, Bender HS, Kheradpour P, Rebbeck CA, et al. The Tasmanian devil transcriptome reveals Schwann cell origins of a clonally transmissible cancer. Science (80- ). 2010/01/02. 2010;327: 84–87. doi:10.1126/science.1180616
3. Caldwell A, Coleby R, Tovar C, Stammnitz MR, Kwon YM, Owen RS, et al. The newly-arisen Devil facial tumour disease 2 (DFT2) reveals a mechanism for the emergence of a contagious cancer. Elife. 2018;7: e35314. doi:10.7554/eLife.35314
