# Supplementary material for: The differentiation state of the Schwann cell progenitor drives phenotypic variation between two contagious cancers
Source: PLoS Pathog. 2021 Nov 15;17(11):e1010033. doi: 10.1371/journal.ppat.1010033 (PMC8629380; doi:10.1371/journal.ppat.1010033)
Supplement: Attachment — Submitted filename: Response to Reviewers_Owen et al.docx [file ppat.1010033.s013.docx]

**Owen et al - Response to Reviewers**

**Reviewer #1:**

In the Owen et al manuscript, the authors describe the Schwann cell origins of both lineages of Tasmanian devil facial tumour disease (DFT1 and DFT2). Through proteomic analysis, they show that the two cancer lineages are most like Schwann cells of different levels of differentiation (with DFT1 the most “differentiated”). This is an interesting report, and while it doesn’t have a huge impact, it does provide important insights into transmissible cancer and it is a fitting topic for PLOS Pathogens. I have several minor comments and two more major concerns about data.

1. The data in Fig 2 are described as showing a time course in which DFT2 expression is responsive to axonal signals, but I can’t see anything that looks like a difference over time in the data. The data are plotted as though it were qRT-PCR data, with levels of expression, but this appears to be just RT-PCR, not qRT-PCR. It is unclear why that was done, as the data cannot be used to make quantitative comparisons. It is unclear if fluctuation from medium-light-pink to light-light-pink and back is anything other than a small amount of noise in the data (there is no explanation of what those different pinks represent in the methods). As this is not qRT-PCR, there is no scale and no sense of the standard errors of the data, this is hard to use to draw any conclusions about responsiveness. Based on the gels in Figure S1, you can make an argument for a clear decrease in MPZ in DFT2 and NGFR in DFT1, as well as an odd fluctuation on NGFR in DFT2. Was this RT-PCR replicated or only done once? In the absence of qRT-PCR data, the actual gel images should probably be used instead of the pink-map, which seems to obscure the larger trends visible in the gel. The control lanes do clearly show differences between DFT1 and DFT2 expression levels, as described.

*We agree with the reviewers points here in regard to the controls and there is a fundamental issue of the use of media conditioned by mouse neurons (see Reviewer 2). It is difficult to adequately control this experiment without the use of either Tasmanian devil Schwann cells or conditioned media from Tasmanian devil neurons, both of which are not possible to currently derive. Even with additional replicates, quantitative RT-PCR and statistical analysis, this fundamental issue remains. For these reasons we have removed this data. However, the differential protein expression patterns found between DFT1 and DFT2, in conjunction with the contrasting response of DFT1 and DFT2 to inflammatory signals, demonstrates that DFT2 has derived from a de-differentiated Schwann cell when compared to the differentiated state of DFT1.*

2.      In figure 4, the DFT1 flow data should definitely be shown, as the authors are claiming that DFT2 is more responsive than DFT1. Just showing a p-value without the data is not enough.

*We thank the reviewer for this point and we have included the data as histograms (this can now be found in* ***Figure 3, panel A****). P values are shown in Panel B.*

3.	The second sentence in the abstract is not correct. Two lineages of bivalve transmissible neoplasia have also been found arising from cockles (C. edule) and mussels (M. trossulus). It is an easy mistake to make, as one of the lineages from M. trossulus has spread to two other Mytilus species, so it correctly summarized as 6 reported bivalve transmissible neoplasia lineages in 6 species, although it is not one-to-one. This doesn’t chance the importance of the findings of the paper, but it just can’t be said that DFTD is the only case of two lineages in a species.

*We thank the reviewer for raising this point and we have now specified that Tasmanian devils are the only mammalian species to carry two lineages* ***(page 2, line 17-18)****.*

4.      There appears to only be a single DFT1 line tested, while 3 DFT2 lines were tested. The paper would be made more solid with additional DFT lines.

*Only one DFT1 line was used in these experiments as MHC-I expression in response to IFNγ treatment in these cells has been previously defined in Siddle et al. PNAS (2013) and we saw no need to replicate this data. In that paper a small amount of variation in MHC-I expression was observed between three DFT1 cell lines, but it was not statistically significant. Thus, we are confident that DFT1_4906 is representative of the DFT1 cell lines commonly used to define DFT1 responses. In the current work we have predominantly focussed on direct comparison between DFT2_RV and DFT1_4906, but we have included the response of DFT2_SN and DFT2_549 to IFNγ in* ***Figure 3, panel A****, demonstrating a consistent response between the three DFT2 cell lines when compared to DFT1.*

3.      Figure 4. Please label DFT1 and DFT2.

*DFT1 and DFT2 have now been labelled in all panels of* ***Figure 3****.*

4.      The limitations of using cell lines rather than primary samples should be mentioned.

*This is a very valid point and an ongoing issue for DFT work, we acknowledge while the cell lines are useful they may not represent the primary tumours currently circulating in the population. We have now raised this limitation in the discussion* ***(page 11, from line 249)****. In addition, we have included staining of Tasmanian devil sciatic nerve and primary DFT1 and DFT2 tumours to extend our analysis* ***(Figure 4 and page 9 from line 202)****. We have limited this staining to antibodies validated in this species as many human/mouse antibodies do not accurately detect devil proteins.*

5.      I would be interested in more speculation about whether this more differentiated state in DFT2 would make a cancer better able to transmit or more likely to metastasize. Since DFT2 is newer it seems it is difficult to make a direct comparison of infectivity, but is anything known about the rates of metastasis between DFT1 and 2?

*This is a very interesting point and we have now addressed this point further in the discussion section* ***(page 13, from line 325)****. There is very little information on metastasis of DFT1 and DFT2, primarily as animals are rarely euthanised due to conservation considerations. However, we consider transmission between individuals akin to metastasis and as DFT2 becomes more established in the population (which seems highly likely) it should be possible to detect whether DFT2 is more transmissible due to its dedifferentiated phenotype.*

6.      Are there any markers which could distinguish a pre-differentiation Schwann cell and de-differentiated repair cell? The authors mention that repair cells have “a distinct phenotype” (page 4 line 75), but don’t explicitly say whether DFT2 was one or the other. The “neuron and repair Schwann cell marker Microtubule Associated Protein 1B (MAP1B)” is higher in DFT2—is this a specific marker of this or not? Some parts of the discussion seem to suggest a repair phenotype, but I didn’t see any direct claim that the study had determined which was the source. If it cannot be determined, that is fine, but it should be explicitly mentioned that those possibilities could not be distinguished.

*There is no single marker that distinguish a pre-differentiation and de-differentiation repair cell (Jessen and Murphy, 2016; Boerboom et al., 2017 (full citations in manuscript)). MAP1B is a protein marker associated with the repair Schwann cell rather than an immature Schwann cell, but it is also expressed in other cell types, as are many of the markers discussed here, making it difficult to link the expression of a single marker to a cell type of origin. However, the overall protein expression and ontology profile, combined with MHC-I expression and inflammatory responses of DFT2 cells is certainly consistent with a de-differentiated Schwann cell in a repair-like state. We have now explicitly stated in the discussion that it is difficult to determine whether DFT2 has emerged from a repair or an immature Schwann cell* ***(page 10, from line 243)****. In saying this, the physiological niche that an immature and de-differentiated Schwann cells occupy is very different and ‘given that immature Schwann cells are a developmental state which are not widely seen in adult nerves, our data suggests that DFT2s emergence from a repair Schwann cell following nerve injury is a likely mechanism of tumour emergence’* ***(page 10-11, lines 246 – 249)****.*

**Reviewer #2:**

Owen and colleagues’ study uses proteomics and RTPCR to compare the gene expression profiles of the two Tasmanian devil contagious cancers, DFT1 and DFT2. Although the study addresses interesting questions, I have concerns about the robustness of the presented data and the validity of the conclusions drawn.

Owen et al present three sets of experiments, as summarised below. I have added my concerns below within the discussion of each experiment.

1. Using proteomics, they search for differentially expressed genes between DFT1 and DFT2 and between representative cell lines of each cell type and fibroblasts. Their results confirm recent work by Patchett et al 2020 suggesting that DFT2, like DFT1, arose from the Schwann cell lineage. Based on the differences in gene expression between DFT1 and DFT2, Owen et al additionally conclude that DFT2 arose from a less differentiated precursor cell than DFT1.

The proteomics analysis itself is clear and well-supported and the results nicely described. However, the authors do not seem to have considered an alternative interpretation for the differences in gene expression between DFT1 and DFT2. Isn’t it possible that DFT1 and DFT2 arose from very similar precursor cells, but that their gene expression profiles diverged during the process of carcinogenesis and/or cell line establishment? I’m not disputing that Owen et al’s interpretation may be correct, but I believe that this alternative explanation should also be considered.

*This is an interesting point and we have now raised this possibility in the discussion section* ***(page 10, from line 236)****. However, high expression of myelin components by DFT1 is strong evidence for emergence from a differentiated cell type, as myelination is associated with growth cessation in Schwann cells and thus they’re unlikely to be upregulated during oncogenesis. This expression profile is highlighted in the protein expression and ontology data in* ***Figure 1*** *and* ***Figure 2, panel A****. While it is possible that DFT2 emerged from a myelinating phenotype that has lost expression of myelin components, the high expression of multiple immature and repair cell markers including MAP1B, L1CAM, NGFR and FABP7 (****Figure 1, panels C and D; Figure 2, panel A****) collectively is unlikely to have occurred entirely during oncogenesis, suggesting these markers were present in the original cell type. Additionally, the expression of MHC-I by DFT2 cells and primary tumours, whilst variable, is not consistent with emergence from a mature myelinating Schwann cell which are largely classical MHC-I negative* ***(Figure 4)****, and for a transmissible tumour, MHC-I expression is an obvious evolutionary disadvantage which is unlikely to have emerged as a result of oncogenesis.*

2. In the second set of experiments, Owen et al expose DFT1 and DFT2 cell lines to neuron-conditioned media and measure the expression of Schwann cell differentiation markers every 24 hours for 7 days. Their hypothesis is that the DFT2 cell line may be more responsive to neural signals due to its precursor-like cell state. They observe that gene expression appears to alter more in DFT2 during the experiment than in DFT1, seemingly supporting the hypothesis.

However, to me this experiment lacks appropriate controls and replicates, and, in its current state, I do not think that the results can be accurately interpreted. It appears that gene expression has been measured only once, in just one cell line of each type. How representative are these measurements?

Further, untreated controls are only represented for a single time-point, not for the full course of the experiment.

The authors have used conditioned media from mouse neurons, is there any expectation that mouse neural growth factors would be functional in Tasmanian devils?

*We agree with the reviewers points here in regard to the controls and the use of media conditioned by mouse neurons. It is difficult to adequately control this experiment without the use of either Tasmanian devil Schwann cells or conditioned media from Tasmanian devil neurons, both of which are not possible to currently derive. For these reasons we have removed this data. The proteomics and the contrasting response of DFT1 and DFT2 to inflammatory signals more adequately supports the argument that DFT2 has derived from a de-differentiated Schwann cell.*

3. In the third set of experiments, the authors assess response to recombinant IFNgamma in one DFT1 and three DFT2 cell lines. They report changes in expression in myelin-related and immune-related genes in the presence and absence of IFNgamma, and conclude that their observations support the idea that DFT2 emerged from a more precursor-like cell than DFT1.

Similar to point (2), it appears that gene expression has been measured only once for each cell line, so the representativeness of the findings is unclear, although in these experiments the consistent inclusion of a no-treatment control is welcome.

*We thank the reviewer for raising this point and we have strengthened this data by repeating the experiment with three biological replicates* ***(Figure 2 and Figure 3)****.*

Overall, although I applaud the authors’ efforts to address an interesting question in a challenging system, I am left unconvinced that the paper’s central message “The differentiation state of the Schwann cell progenitor drives phenotypic variation between two contagious cancers” is in fact well-supported.

*Based on the upon the reviewers suggestions above, we have made significent improvements to the manuscript and present convincing evidence that DFT1 and DFT2 derived from Schwann cells in different differentiation states. The proteomics data clearly shows differentiation between the cell types, in particular at markers involved in Schwann cell differentiation and myelination. The response to the inflammatory cytokine, IFNγ, further supports these differences and underlies the ability of DFT1 to respond to this cytokine in comparison to DFT2. We have strengthened this analysis by comparing expression of MHC-I and periaxin in primary DFT1 and DFT2 tumour samples and Tasmanian devil sciatic nerve samples, showing the similarity of DFT1 to differentiated Schwann cells not just in myelination, but in MHC-I expression as well. In addition, we have shown that PRC2, a key transcriptional silencer of repair Schwann cell genes which is involved in differentiation to a myelinating phenotype is expressed in DFT1 cells, but not in DFT2 cells. PRC2 has also been shown to silence MHC-I expression, and thus it provides a link between the differentiation state of the Schwann cell progenitor and their MHC-I expression, providing evidence that it is the differentiation state of the Schwann cell progenitor cells which has directed their molecular phenotype and immune evasion mechanisms.*
