# Supplementary material for: The differentiation state of the Schwann cell progenitor drives phenotypic variation between two contagious cancers
Source: PLoS Pathog. 2021 Nov 15;17(11):e1010033. doi: 10.1371/journal.ppat.1010033 (PMC8629380; doi:10.1371/journal.ppat.1010033)
Supplement: Attachment — Submitted filename: Response to reviewers_Owen et al.docx [file ppat.1010033.s014.docx]

Reviewer #1: In the Owen et al manuscript, the authors describe the Schwann cell origins of both lineages of Tasmanian devil facial tumour disease (DFT1 and DFT2). It had previously been shown that both lineages arose from Schwann cells, but this report goes further to show that one lineage is more differentiated than the other (DFT1 is more differentiated). Notably, this has an effect on the immune evasion strategies of these cancers, as the differentiation state affects MHC-I expression level. The authors have answered all of the reviewers' questions, removing a problematic section and adding significantly improved discussion and analysis of proteomic data.

***Response:***

*We thank the reviewer for the constructive comments on the manuscript.*

Reviewer #2: This manuscript compares the differentiation state of Schwann cells that gave rise to two independent transmissible cancers in Tasmanian devils - DFT1 (the original lineage, described in the 1990s) and DFT2 (described in 2016). Although the gross morphology of tumors on the face of Tasmanian devils is quite similar, DFT1 and DFT2 present differently in terms of histology, and genomic differences have been identified between the two tumors. The cells of DFT1 and DFT2 also have differing levels of MHC expression, as well as responses to IFN-gamma treatment. The experiments herein, primarily a proteomics analysis, show that DFT2 emerged from a less differentiated Schwann cell than DFT1. I have not seen a previous version of the MS and note that the authors have done a good job responding to previous comments and removing less well-supported data. My main concern is that this manuscript is quite specific relating to Tasmanian devils and DFTD, and as written, may not be of general interest to the broad readership of PLoS Pathogens. As such, my general recommendation is to introduce the manuscript in a more broad way (which is done to some extent in the discussion) as to the implications for understanding the differentiation state of Schwann cell tumors. The authors mention the plasticity of Schwann cells, and perhaps a more general discussion as to how this relates to them becoming cancerous would enhance the introduction.

***Response:***

*We thank the reviewer for these helpful comments on the scope of the manuscript. We have addressed these comments by re-writing parts of the introduction to give a background on why cell type of origin is so central to cancer plasticity, highlighting this importance not just in cancers and contagious cancers, but also for oncovirus induced cancers (page 4-5, lines 102 – 129). We have also empahsised the importance of cell type of origin in predicting the evolutionary trajectory of a disease and the general importance of understanding pathogens that are emerging and circulating in our environment (even if they do not infect humans). Finally, we have broadened the final paragraph of the discussion (page 15, lines 405 – 411).*

Reviewer #3: In the revised version of their paper, Owen et al document (1) whole-proteome differences in protein expression between representative DFT1 and DFT2 cell lines, (2) alterations in transcript abundance for selected genes in representative DFT1 and DFT2 cell lines cultured in the presence and absence of recombinant IFNg, (3) differences in the magnitude of upregulation of cell-surface B2M following IFNg treatment among several DFT2 cell lines as well as a DFT1 cell line, and (4) using immunohistochemistry, assess expression of SAHA UABC and UK in Tasmanian devil sciatic nerve and in representative DFT1 and DFT2 tumour biopsies.

In the revision, the authors have performed replicates of the qPCR experiments, and these are now considerably more robust. However, I struggle to follow a coherent story in the paper. Much of the work repeats findings already in the published literature (Patchett et al 2019, Ong et al 2021, Flies et al 2016, Siddle et al 2013, Caldwell et al 2018). The main new contribution is the proposal that DFT1 and DFT2 arose from different progenitor cell states. However, this was already hinted at in Patchett et al 2019, and, in my opinion, despite the authors’ rebuttal, I do not believe that these cancers’ progenitor cell states can be inferred from the presented work. The PCR data presented in figures 2C, 2D, 3C, 3D, S1, S2 detect subtle changes and statistical significance level does not seem to have been corrected for multiple testing. I am left unsure that differences are significant and reproducible. Furthermore, fig 3B shows that there is significant variation in cell surface B2M level among DFT2 cell lines; this makes me wonder how many of the findings from the rest of the paper are specific to the single representative cell lines selected for DFT1 and DFT2.

Overall, I don’t find the results a compelling-enough contribution to the field to warrant publication in PLOS Pathogens.

***General response:*** *We would argue that our data is both highly novel and significant as recognised by the other reviewers. This study represents a detailed examination of two independent lineages of a contagious tumour that have arisen from distinct differentiation states of Schwann cells and how they respond to inflammatory cues. We show that each lineage has adopted novel immune evasion strategies that have facilitated the spread of DTF1 and DTF2 into the wild Tasmanian Devil population. We recognise that this species is not as experimentally tractable to allow the sorts of nuanced experiments that could be performed in laboratory strains of mice, but believe the evidence of their different differentiation states is compelling and provides significant insights into contagious tumours that have arisen and may arise in other species.*

***Specific Responses:***

‘The PCR data presented in figures 2C, 2D, 3C, 3D, S1, S2 detect subtle changes and statistical significance level does not seem to have been corrected for multiple testing. I am left unsure that differences are significant and reproducible.’

*This is a vaild issue and we have now included in the manuscript an explanation of how multiple testing correction wasn used in the proteomics analysis and specific reasons this was not applied to the qPCR experiments (lines 468 - 470 and 518 – 523).*

*The qPCR experiments were treated differently from the proteomics analysis as multiple testing correction is overly stringent for planned experiments with a rationale defined by previous studies (Noble, 2009, Nature Biotechnology; Feise, 2002, BMC Med Res Methodol.;Rothman, 1990, Epidiemiology). In these experiments it is not appropriate to apply these corrections as the biological cotext is important and correction can easily introduce type II errors. In our experiments we selected genes for anaylsis that were predicted to control or define Schwann cell differentiation. The selection of these genes was based on the proteomics data, in which multiple testing correction was applied, and extensive literature on Schwann cell biology covering human, mouse and rat cells. In addition, where the effect in biological replicates is internally consistent (ie. the gene always goes up or down) it is argued that multiple testing is obsolete. We have provided data on our biological replicates below, demonstrating internal consistency (Blue – DFT1, Red – DFT2, note the presentation of this data is for review only, but raw data can be found in S9 Table). Finally, it is not standard practice to correct for multiple testing in qPCR experiments where the number of comparisons low (limiting alpha inflation and the number of false positives ie. 0.05 x 8 = 0.4 incorrectly identified positives) and there is a biological context to the experiments (Yuan et al., 2006, BMC Bioinformatics).*

*Despite these reservations we have carried out a post-hoc Benjamini analysis, which does support significant differences between the cell lines at all the genes in Fig 2, supporting the primary conclusion of the paper, that DFT2 derived from a de-differentiated Schwann cell. L1CAM and PLP1 remain significantly up and down regulated in DFT1 with IFNγ treatment (Fig 2C), while significance in the other genes is lost. However, for the reasons outlined above we have concerns that reporting these results would include type II error (particularly as our biological replicates are consistent).*

‘Furthermore, fig 3B shows that there is significant variation in cell surface B2M level among DFT2 cell lines; this makes me wonder how many of the findings from the rest of the paper are specific to the single representative cell lines selected for DFT1 and DFT2.’

*There is variation in MFI between the DFT2 cell lines, but this is not a key finding of this work and would be better explored in further studies. In this experiment we wanted to test whether DFT1 and DFT2 respond differently to IFNγ as Schwann cell differentiation state is known to affect the response to inflammation. Thus, the aim of Fig 3 is to show that IFNγ induces B2m upregulation in DFT2 cells (although not as marked as DFT1) and that DFT1 and DFT2 have different responses at other Schwann cell markers.*

*We agree with the reviewer that there is a broader issue here of drawing conculsions from in vitro experiments and we have mitigated this to an extent by also including data from biopsies (Fig 4) and mimimising passage of DFT2 and DFT1 cell lines. Finally, we have discussed this limitation in the discussion section (page 11, line 310-312) and if DFT2 continues to spread it is likley more DFT2 biopsies will become available for analysis.*

L1CAM

Expression relative to RPL13A

| Biological replicate | Control | Treated |
| --- | --- | --- |
| 1 | 0.0706619 | 0.231159 |
| 2 | 0.118443 | 0.213625 |
| 3 | 0.10983 | 0.159116 |

PRX

Expression relative to RPL13A

| Biological replicate | Control | Treated |
| --- | --- | --- |
| 1 | 1.30674 | 0.213676 |
| 2 | 1.26242 | 0.677152 |
| 3 | 1.01135 | 0.934718 |

PMP22

Expression relative to RPL13A

| Biological replicate | Control | Treated |
| --- | --- | --- |
| 1 | 1.08497 | 0.902527 |
| 2 | 1.33348 | 0.623958 |
| 3 | 0.917998 | 0.581318 |

PLP1

Expression relative to RPL13A

| Biological replicate | Control | Treated |
| --- | --- | --- |
| 1 | 1.34685 | 0.494556 |
| 2 | 1.48683 | 0.543014 |
| 3 | 1.10345 | 0.766808 |

PMP22

Expression relative to RPL13A

| Biological replicate | Control | Treated |
| --- | --- | --- |
| 1 | 0.0473029 | 0.0415924 |
| 2 | 0.0509758 | 0.0355479 |
| 3 | 0.0506648 | 0.0293148 |
